# Mechanosensitive ion channels as novel targets in osteoporosis

**DOI:** 10.1093/jbmr/zjaf145

**Published:** 2025-12-10

**Authors:** Christoph Beyersdorf, Uwe Maus, Felix Wiedmann, Juliana Franziska Bousch, Maximilian Waibel, Constanze Schmidt, Merten Prüser

**Affiliations:** Department of Orthopaedics and Trauma Surgery, Medical Faculty, Heinrich Heine University Duesseldorf, 40225 Duesseldorf, Germany; Department of Orthopaedics and Trauma Surgery, Medical Faculty, Heinrich Heine University Duesseldorf, 40225 Duesseldorf, Germany; Department of Cardiology and Pneumology, University Medical Center Goettingen, 37075 Goettingen, Germany; German Center for Cardiovascular Research (DZHK), Partner Site Lower Saxony, Georg-August-University Goettingen, 37075 Goettingen, Germany; Department of Internal Medicine III, Medical University Hospital Heidelberg, 69120 Heidelberg, Germany; German Center for Cardiovascular Research (DZHK), Partner Site Heidelberg/Mannheim, University of Heidelberg, 69120 Heidelberg, Germany; Department of Orthopaedics and Trauma Surgery, Medical Faculty, Heinrich Heine University Duesseldorf, 40225 Duesseldorf, Germany; Department of Internal Medicine III, Medical University Hospital Heidelberg, 69120 Heidelberg, Germany; German Center for Cardiovascular Research (DZHK), Partner Site Heidelberg/Mannheim, University of Heidelberg, 69120 Heidelberg, Germany; Department of Cardiology and Pneumology, University Medical Center Goettingen, 37075 Goettingen, Germany; German Center for Cardiovascular Research (DZHK), Partner Site Lower Saxony, Georg-August-University Goettingen, 37075 Goettingen, Germany; Department of Internal Medicine III, Medical University Hospital Heidelberg, 69120 Heidelberg, Germany; German Center for Cardiovascular Research (DZHK), Partner Site Heidelberg/Mannheim, University of Heidelberg, 69120 Heidelberg, Germany; Department of Internal Medicine III, Medical University Hospital Heidelberg, 69120 Heidelberg, Germany; German Center for Cardiovascular Research (DZHK), Partner Site Heidelberg/Mannheim, University of Heidelberg, 69120 Heidelberg, Germany

**Keywords:** osteoporosis, osteoblasts, macrophages, osteoimmunology, inflammation, ion channels

## Abstract

Osteoporosis is the most prevalent metabolic bone disease globally, leading to an increased risk of fractures. Recent advances in ion channel research have shed light on the importance of mechanosensitive ion channels as novel players in these pathophysiological processes. This perspective discusses the involvement of the mechanosensitive ion channels TREK-1, Piezo, and volume-regulated anion channels (VRACs) as potential novel pharmacological targets for the treatment of osteoporosis. TREK-1, a mechanosensitive K_2P_ channel is important for maintaining the resting membrane potential in many cells, including osteoblasts and osteoclasts. K_2P_ channels regulate osteoblast proliferation and differentiation, as well as osteoclast activity, potentially modulating bone remodeling in osteoporosis. Piezo channels influence osteoblast differentiation and osteoclast activity by modulating calcium influx, which is crucial for osteogenic signaling pathways, such as Wnt/β-catenin and ERK1/2. Piezo1 activation promotes bone formation, while its deficiency leads to impaired osteogenesis and increased bone resorption. Volume-regulated anion channels have been shown to be involved in osteoblast adaptation to mechanical stress and macrophage polarization, which indicates their importance for bone homeostasis. Chronic inflammation is a major contributor to osteoporosis progression. Evidence of ion channel involvement in this process has emerged in recent years. Specifically, macrophage function in osteoporosis seems to be linked to ion channel activity. Inflammatory polarization of macrophages is a key player in inflammation-induced bone loss and can be driven by mechanosensitive ion channels. Modulating these ion channels may provide new therapeutic opportunities. Given the complexity of ion channel interactions in bone cells and their regulatory role in bone remodeling, understanding their precise function in osteoporosis is essential. Targeted modulation of mechanosensitive ion channels holds promise as a novel therapeutic approach to mitigate inflammation-driven bone loss and improve bone density. Further research into their role in osteoclasts and macrophage-driven bone degradation will aid in developing innovative osteoporosis treatments.

## Graphical Abstract

**Graphical Abstract:**
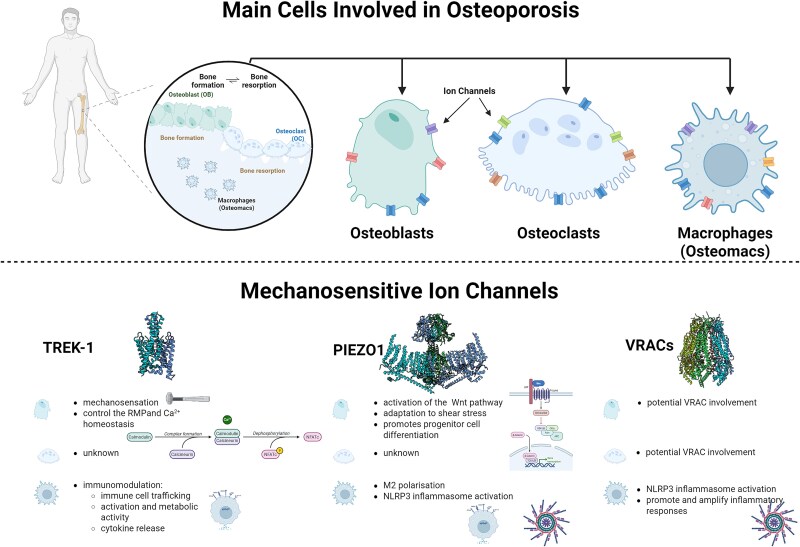

## Introduction

Osteoporosis, the most common metabolic bone disease globally, is characterized by reduced bone mass and microarchitectural deterioration of bone tissue. This, in turn, leads to increased susceptibility to fractures, posing a significant public health challenge, particularly among aging populations.^1^ While the etiology of osteoporosis is complex and multifactorial, encompassing genetic predisposition, hormonal changes, and lifestyle factors, at its core there is an imbalance of osteoblastic bone formation and osteoclastic bone resorption. The precise molecular mechanisms underlying this disruption of physiological bone homeostasis, including the involvement of ion channels; however, remain incompletely understood.

Since the advent of “osteoimmunology” as a distinct field in the year 2000, the relationship between inflammatory processes and bone metabolism has gained increasing attention.^2^^,^^3^ It is now well established that inflammation promotes bone resorption. Chronic low-grade inflammation associated with aging (termed “inflammaging”) and systemic inflammatory diseases, such as rheumatoid arthritis, exacerbate bone loss, and contribute to the progression of osteoporosis.^4^

Recent advances have highlighted the pivotal role of osteal macrophages (Osteomacs) in bone remodeling. These specialized macrophages predominantly originate from embryonic tissue, specifically the yolk sac or fetal liver, and are capable of self-renewal independent of circulating monocytes, adapting their functions to the respective environment.^5^ While it was initially not possible to distinguish Osteomacs from bone-marrow-derived macrophages (BMDMs), it is now known that they exhibit phenotypically and functionally distinct characteristics.^6^ Osteomacs are closely associated with bone surfaces and actively regulate the activities of osteoblasts and osteoclasts.^7–9^ A recent study by Batoon and colleagues was the first to highlight the involvement of Osteomacs in the pathophysiology of osteoporosis.^8^ Using a murine model of postmenopausal osteoporosis, the researchers demonstrated that Osteomacs are closely associated with osteoclasts. They hypothesized that these macrophages facilitate bone resorption by phagocytosing osteoclast-derived byproducts. Despite these findings, the broader role of Osteomacs in osteoporosis remains poorly understood, particularly regarding their potential contribution to inflammation-driven bone loss.

Macrophages are highly versatile immune cells characterized by a high degree of plasticity. Depending on the appropriate environmental cues, they can polarize into distinct functional phenotypes, including the pro-inflammatory M1 type and the anti-inflammatory M2 type.^10^ While these classical dichotomies offer a simplified framework, advances in single-cell RNA sequencing have revealed a more complex spectrum of macrophage phenotypes with unique transcriptional signatures and functional roles.^11^ The environmental conditions and molecular mechanisms leading to a distinct polarization are incompletely understood, but ion channels, especially Piezo1, have been reported to be heavily involved.^12^

A critical regulator of macrophage-driven inflammation is the NLRP3 inflammasome, a multiprotein complex that mediates the activation of caspase-1 and the subsequent release of pro-inflammatory cytokines IL-1β and IL-18.^13^ Elevated NLRP3 activity has been implicated in both bone resorption and impaired bone formation, suggesting a direct link to the pathogenesis of osteoporosis.^14^

Bone homeostasis relies on the delicate balance between the activities of osteoblasts, osteoclasts, and immune cells, such as Osteomacs. This critical regulatory equilibrium is often disrupted during inflammatory conditions. Although significant research in this field has been conducted, developing therapies to address inflammation-induced bone loss continues to pose a considerable challenge.

While much attention with regards to pharmacological intervention has focused on cytokines and signaling cascades, ion channels are increasingly recognized as important regulators of inflammation in bone cells, for example, by integrating mechanical and inflammatory cues.^15^ Moreover, ion channels have, for decades, been regarded as easy pharmacological targets, which is why, even today, they are the second most important pharmacological targets after G-protein coupled receptors and have been estimated to account for approximately 20% of all available drugs. Despite their pathophysiological and pharmacological significance, the role of ion channels in the pathogenesis of osteoporosis remains an underexplored area of research.

This perspective aims to highlight the intricate relationship between three presently understudied mechanosensitive ion channels and osteoporosis, especially in osteoblasts, osteoclasts and macrophages, exploring their functions, mechanisms, and therapeutic potential. This article does not intend to provide a comprehensive overview of all the ion channels involved in bone metabolism. While it is recognized that other ion channels, especially those of the TRP family, are critically involved in bone metabolism,^16^ they were not the focus of this perspective. Instead, we chose to highlight less well-known but conceptually promising ion channels whose diverse biophysical properties—such as activation by membrane stretch, shear stress, or osmotic cell swelling—and dual regulators of mechanical and inflammatory signaling, make them attractive candidates for future therapeutic strategies in osteoporosis. Specifically, we will discuss three key families of ion channels, the mechanosensitive Piezo and K_2P_ channels, as well as the volume-regulated anion channels (VRACs). These channel classes were discovered fairly recently, with known Cryo-EM structures and pharmacological modulators, but have not been well studied in the context of bone diseases, thus making them interesting novel targets for pharmacological intervention to treat osteoporosis.

## Two-pore-domain potassium (K_2P_) channels

In 1995, Ketchum and colleagues were the first to describe the existence of outwardly rectifying background potassium channels “with two pore domains in tandem”, which were cloned from *Saccharomyces cerevisiae* (TOK-1) and are thought to have evolved from duplication of K_ir_ channel genes. They operate across the physiological voltage range and maintain the resting membrane potential in both non-excitable and excitable cells, as well as modulate the shape of the action potential in excitable cells. A total of 15 members of the K_2P_ family have been identified.^17^ Of these, 12 are known to produce currents in native cells or heterologous expression systems. However, three K_2P_ channels, TASK-5, TWIK-3, and THIK-2, have not been shown to generate macroscopic currents. Recent studies suggest that these three α-subunits form heteromeric channels with other K_2P_ subunits, playing a crucial role in controlling their targeting to the membrane surface.^18^^,^^19^

**Figure 1:**
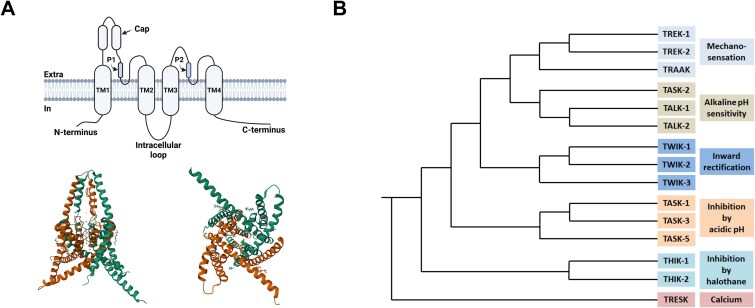
(A) Upper left: structure of a K_2P_ channel subunit (created with BioRender). Lower left: crystal structure of a homodimeric TREK-1 channel (PDB: 4TWK^*^, left: side view, right: bottom view). (B) Dendrogram of the K_2P_ channel family (modified after,^48^ created with BioRender).

K_2p_ channels have a distinct 2×2 topology: 2 α-subunits with 2 pore domains each form active pseudotetrameric channels with asymmetrical pores (other K^+^ channels consist of 4 α-subunits with 1 pore domain each and, thus, have symmetrical pores; see Figure 1). They share comparatively low sequence homology within their family and are subclassified mainly according to functional characteristics, such as acid-sensitivity, inhibition by halothane, etc. In addition to forming homomers, K_2p_ channels have been demonstrated to form heterodimeric channels with other members of the K_2P_ family, which increases their functional diversity and may confer resistance to pharmacological agents. They are modulated by a wide range of stimuli and regulatory mechanisms, such as lipids, neurotransmitters, membrane voltage, mechanical stretch, fluctuations in temperature, as well as changes in pH, and are expressed in nearly all living cells. In recent years, interest in pharmacological modulation of these channels to treat diseases, such as sleep apnea and atrial fibrillation, has risen significantly.^20^^,^^21^

In the context of bone biology, K_2P_ channels have been suggested to regulate both osteoblast proliferation and osteoclast activity. Extracellular acidosis, a hallmark of active bone resorption, inhibits K_2P_ channel activity in osteoblast-like MG-63 cells, thereby reducing their proliferation.^22^ Similarly, potassium channel mediated membrane hyperpolarization has been identified as a critical regulator of osteogenic differentiation in skeletal progenitor cells.^23^

In osteoclasts, there is, at present, mostly indirect evidence of K_2P_ channel regulation. Extracellular acidosis in these cells, for example, has been shown to promote differentiation and activity via calcium-mediated NFATc1 activation.^24^ G-protein-coupled receptors (GPCRs), such as ovarian cancer G protein-coupled receptor 1 (OGR1) and T-cell death-associated gene 8 (TDAG8), play an important role in acid-sensing in osteoclasts.^25^ It is also known that non-GPCRs are also involved in acid-sensing. Transient receptor potential V1 and acid-sensitive ion channels appear to play a role in osteoclasts in this context.^26^ Further downstream of that, a calcium-mediated nuclear translocation of NFATc1 seems to be involved in both GPCR and non-GPCR signaling.^24^ The acid sensitivity of some K_2P_ channels and shared lineage with macrophages suggests functional relevance. Indeed, Yeon et al. postulated a negative regulatory role of the K_2P_ channel TWIK-1 in osteoclastogenesis through inhibition of calcium oscillations and the JNK-NFATc1 signaling cascade.^27^ The role of K_2P_ channels in macrophages is well-documented, which further supports their potential involvement in osteoclasts as well.^28^ However, it should be noted that this assumption warrants verification in follow-up studies, as osteoclasts and macrophages differ substantially in their gene expression profiles and functional specialization.

Of note, Pangalos and colleagues showed that osteoblasts are excitable cells that generate repetitive action potentials after application of a depolarizing stimulus.^29^ It was postulated that the ability to generate action potentials may be involved in osteoblast differentiation during osteogenesis. Significant differences in the ability to generate action potentials were reported between osteoporotic and healthy cells. The exact physiological significance of action potentials in osteoblasts remains unclear. This highlights that the classic binary classification into excitable and non-excitable cells may be overly simplistic or not always accurate.

We specifically focus on elucidating the role of two-pore-domain potassium (K_2P_) channels because there is strong evidence for an important role in both osteoblasts and macrophages, supporting the assumption that they may also be functionally relevant in Osteomacs. In osteoclasts, available data are sparse; however, both mechanistic considerations and specific evidence for the K_2P_ channel TWIK-1 point to a potential role in this cell type. Given that Osteomacs and osteoclasts are central regulators of bone resorption and remodeling, clarifying the contribution of K_2P_ channels in these cells is of considerable interest. Nonetheless, the current evidence base for Osteomacs and osteoclasts remains limited, and further studies—particularly in vivo—are needed to verify their involvement in bone metabolism. Within the K_2P_ family, TREK-1 emerges as a particularly promising target due to its unique combination of mechanosensitivity and immunomodulatory properties, positioning it at the intersection of mechanical and inflammatory regulation in bone.

### TREK-1

TREK-1 (tandem of a pore-domain in a weakly inward rectifying K^+^ (TWIK) related K^+^ channel 1, also known as K_2P_2.1), a mechanosensitive member of the K_2P_ family, is highly expressed in excitable cells, especially in neurons,^30^ as well as, to a lesser extent, in human cardiomyocytes.^31^ It is sensitive to both membrane stretch and shear stress induced by fluid flow.^32^ Studies on the conformational changes induced by mechanical stress suggest a stabilization of the channel’s high conductive up-state vs the low-conductive down-state.^33^

Hughes and colleagues were able to demonstrate in 2006 that TREK-1 is also expressed in human primary osteoblasts and “osteoblast-like” MG-63 cells.^34^ Functional analysis in whole cell patch clamp experiments demonstrated a constitutionally active outwardly rectifying potassium “leak” current comparable to TREK-1 currents, which are essential for resting membrane potential maintenance. It is known that the resting membrane potential has a decisive influence on the proliferation rate of cells. Therefore, Hughes and colleagues cultured MG-63 cells in the presence of a TREK-1 blocker (Ropivacain). In this study, the proliferation rate increased significantly after administration of Ropivacain. It was therefore postulated that osteoblasts functionally express TREK-1 and that these channels may be involved in mechanotransduction and thus in bone remodeling. Involvement here is conceivable either directly through the perception of mechanical stimuli or as a downstream signaling tool, since some known modulators of mechanotransduction, such as cAMP, NO, and prostaglandins also modulate TREK-1.^34^^,^^35^

Additionally, TREK-1 may influence cellular behavior through calcium concentration changes caused by altered potassium conductance. Calcium is an important second messenger that regulates a variety of cellular functions, such as proliferation, apoptosis, and secretion.^36^ Hyperpolarization of the membrane potential induced by increased potassium conductance can lead to enhanced calcium influx via two mechanisms: first, voltage-independent calcium influx through an increased electromotive driving force for calcium via voltage-independent ion channels and intracellular stores.^37^ Second, through voltage-dependent ion channels that are activated by membrane hyperpolarization,^38^such as HCN channels, which can be expressed in various organs, including in osteoclasts.^39^

Intracellular calcium influences cellular behavior in osteoblasts by modulating calcium-dependent proteins, such as calcium/calmodulin-dependent kinase II, calcineurin (Cn), and calreticulin (CRT). Calcium/calmodulin-dependent kinase II can activate the ERK signaling pathway in osteoblasts,^40^ while Cn can influence bone remodeling by modulating the osteogenic transcription factor “nuclear factor of activated T-cells” (NFAT).^41^ Calreticulin can also affect osteoblast differentiation through the NFAT signaling network.^23^ Hyperpolarization of the membrane potential led to increased osteogenic differentiation in MSCs,^42^ and similarly, inhibition of K_2P_ channels in MG-63 cells resulted in a reduced proliferation rate.^22^

In macrophages, potassium efflux is required for NLRP3-inflammasome activation, although it is not yet clear which channels are involved. Recently, it was shown that TREK-1 is essential for NLRP3 activation in BMDM.^28^ Recent studies have also demonstrated the potential influence of potassium currents on macrophage polarization.^43^ TREK-1 is recognized for its immunomodulatory properties, including the regulation of immune cell trafficking, activation, and metabolic activity, as well as the modulation of proinflammatory cytokine secretion, such as interleukin-6 (IL-6).^44–46^ In contrast, much less is known about how a sustained inflammatory microenvironment—such as that observed in osteoporotic bone—affects TREK-1 activity. Indirect evidence suggests that inflammatory conditions may indeed activate the channel. TREK-1 is sensitive to extracellular pH changes and hypoxia, both of which are common features of inflamed tissues. Furthermore, its activity is regulated via PKC- and PKA-dependent phosphorylation, signaling pathways that play a central role in the inflammatory response.^47^

Although other channels, such as those of the Piezo family, are more directly linked to bone mechanotransduction, the combined mechanosensitive and immunomodulatory properties of TREK-1, together with experimental evidence in osteoblasts and macrophages, suggest that it may represent a promising mechanistic target for future studies aimed at modulating bone resorption and formation in osteoporosis.

## Piezo channels

**Figure 2:**
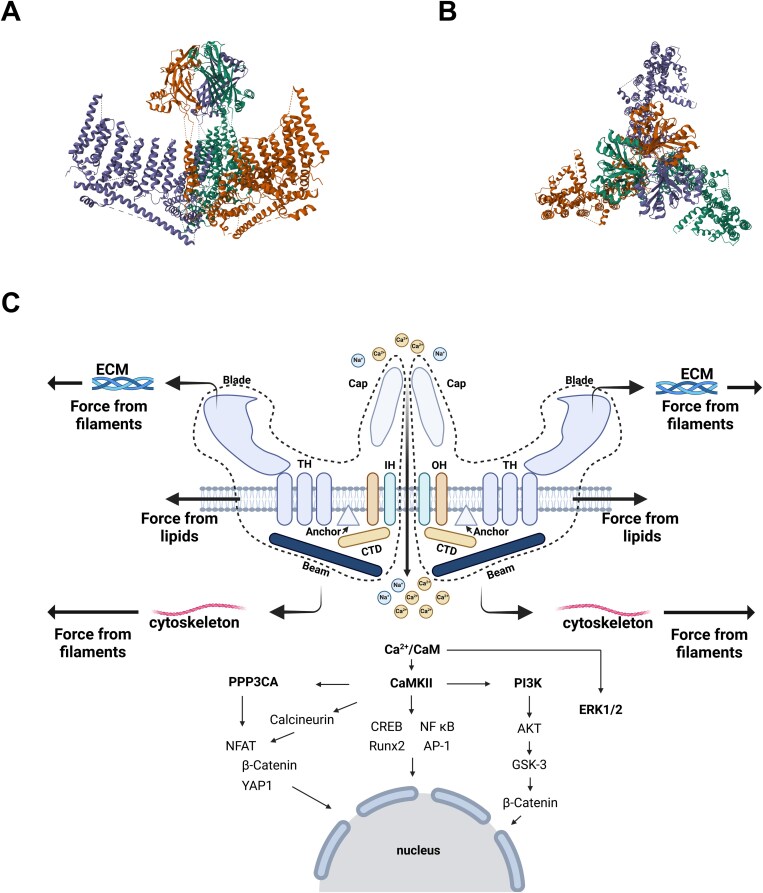
(A) + (B) Structure of a piezo1 homotrimer (PDB: 6BPZ). (C) Schematic diagram of piezo1, including the proposed gating mechanisms and the downstream signaling pathways; IH, inner helix; OH, outer helix; THs, transmembrane helices; CTD, intracellular C-terminal domain, modified after,^53^ created with BioRender.

Piezo channels are large (900 kDa) mechanosensitive proteins with 30-40 transmembrane domains that respond to mechanical stimuli by regulating the influx and efflux of cations, such as calcium (Ca^2+^), magnesium (Mg^2+^), potassium (K^+^), and chloride (Cl^−^)^49^ (see Figure 2). The Piezo family consists of two main channels: Piezo1 and Piezo2, which share 47% sequence homology. Piezo channels are expressed in various tissues, including the vascular system, bladder, skin, and bone. They play a crucial role in numerous physiological processes, such as touch sensation, blood pressure regulation, and, crucially, bone homeostasis.^50^ Osteoblasts and osteocytes are sensitive to mechanical load and strain, and maintain bone homeostasis by adapting to mechanical stimuli. Piezo1 and Piezo2 have been shown to be abundantly expressed in these cells and are essential for mechanotransduction, as well as cell cycle regulation and intracellular signaling.

Mechanistically, it has been hypothesized that changes in membrane tension in response to mechanical stress open Piezo channels—a mechanism referred to as the force-from-lipid (FFL) model. Alternatively, the force-from-filament (FFF) model is proposed, which suggests that Piezo channels are tethered to the cytoskeleton and the extracellular matrix (ECM). Movements of the cytoskeleton or the ECM under mechanical stress are thought to open the channel via mechanical pushing or pulling forces.^51^

Interestingly, Piezo1 and Piezo2 activation has been shown to influence the activity of other mechanosensitive channels, including TREK-1 and TREK-2. Specifically, Piezo1/2 increase the current amplitude of TREK-1 and slow down inactivation, thus highlighting the crosstalk between different types of mechanosensitive ion channels.^52^

In mice, Piezo1 KO prevented bone formation following mechanical stress. The most likely cause was Piezo-dependent activation of the transcription factors YAP1 and TAZ, which, in turn, have been linked to increased expression of Wnt1.^56^ Wnt1 belongs to the evolutionarily conserved pathways that are essential for cell proliferation and polarity. Activation of the Wnt signaling pathway, for example, through Wnt1, is essential for osteoblast activation and bone formation, while inhibition of the Wnt signaling pathway, for example through sclerostin, leads to increased bone resorption.^54^^,^^55^ Piezo1 has also been shown to regulate Runx2 gene expression through the AKT/GSK-3β/β-catenin cascade,^57^ which, in turn, mediates osteoblast adaptation to mechanical fluid shear stress, Moreover, in vitro studies have shown that Piezo1 promotes skeletal progenitor cell differentiation into osteoblasts by enhancing BMP2 expression through the ERK1/2 signaling pathway.^58^ More broadly, the Wnt signaling pathways, the ERK1/2 signaling pathway, as well as the Akt/GSK-3β/β-catenin cascade are all calcium-dependent and are influenced by calcium influx following Piezo activation.^23^ Additionally, Piezo1 facilitates bone formation by mediating the phosphorylation of AKT, which downregulates Sclerostin—a protein that inhibits osteoblast-mediated bone formation through Wnt/β-catenin-inhibition.^59^ Conversely, mechanical unloading inhibits Wnt1 expression by inactivation of Piezo1. Unloading also increases RANKL expression in osteocytes, promoting bone resorption.^60^ In addition to its role in mechanically induced bone formation, Piezo1 is also essential for basal bone formation. Conditional KO of Piezo1 in skeletal progenitors or osteoblasts has been shown to reduce bone mass under normal conditions, highlighting its critical contribution to skeletal development and homeostasis.^61^^,^^62^

Moreover, Piezo-dependent nuclear translocation of YAP1 in osteoblasts also controls the expression of bone matrix proteins, such as collagen types II and IX. Additionally, Piezo1 indirectly influences bone resorption, as osteoclast differentiation depends on the expression of these collagens. However, direct deletion of Piezo1 in mouse osteoclasts did not alter bone mass.^61^

While Piezo2 deletion alone does not produce a detectable bone phenotype in mice, the more severe skeletal defects observed in Piezo1/Piezo2 double KOs indicate that Piezo2 may contribute to bone development and that its apparent dispensability in single KOs likely reflects compensatory activity by Piezo1.^63^ In line with this, Piezo1 expression has been shown to be significantly reduced in human osteoporotic bone.^62^

In macrophages, activation of Piezo1 through mechanical stimulation induced a conversion of macrophages to the M2 phenotype. This polarization shift was not possible after a Piezo1 knockdown using siRNA, suggesting that Piezo1 is essential for M2 polarization of macrophages through mechanical stimulation.^64^ Furthermore, Piezo1 activation in macrophages leads to NLRP3 inflammasome activation. Ran and colleagues recently demonstrated that Piezo1-mediated calcium influx activates a calcium-dependent potassium channel (KCNN4), which subsequently drives NLRP3 inflammasome activation.^65^ Furthermore, macrophages lacking Piezo1 secrete significantly fewer pro-inflammatory cytokines, such as TNFα and IL-6, following inflammatory activation by LPS and INFγ.^12^ These pro-inflammatory effects of Piezo1 activation in macrophages contrast with the observed beneficial effects on bone metabolism described earlier, suggesting a more complex regulatory function in bone homeostasis.

Interestingly, in a pulmonary inflammation model, mechanical activation of Piezo1 exacerbated the inflammatory response. Piezo1-mediated calcium influx activated activator protein-1 (AP-1), promoting the transcription of endothelin-1 (Edn1), which stabilized hypoxia-induced factor 1α (HIF1α). This stabilization, in turn, induced a pro-inflammatory expression profile, including IL-1β and TNFα.^66^ It has also been shown in bone tissue that HIF1α has a regulatory function regarding cytokine secretion and macrophage polarization.^67^

The immunomodulatory function of Piezo channels have been demonstrated in a range of chronic inflammatory diseases, including periodontitis, pulmonary inflammation, atherosclerosis, Alzheimer’s disease, and osteoarthritis.^68^ In these contexts, Piezo channels have been implicated in shaping immune responses, although their precise contribution to inflammation modulation varies between disease models. Notably, inflammatory mediators such as interleukin-1β (IL-1β) can directly affect Piezo channel expression and activity.^69^ In addition, inflammation-induced changes in tissue mechanics—such as edema, ECM degradation, and cytoskeletal remodeling—can indirectly influence Piezo channel gating. While these findings highlight a broad role for Piezo channels in inflammation, their immunomodulatory function in osteoporosis, particularly with respect to osteoblasts and Osteomacs, remains unexplored.

In sum, Piezo1 is arguably the best-studied mechanosensitive ion channel in bone biology, with compelling in vivo evidence for its role in bone formation and homeostasis. Its dual effects on immune cells and bone cells highlight both therapeutic potential and the need to understand cell type-specific responses.

## Volume-regulated ion channels

**Figure 3:**
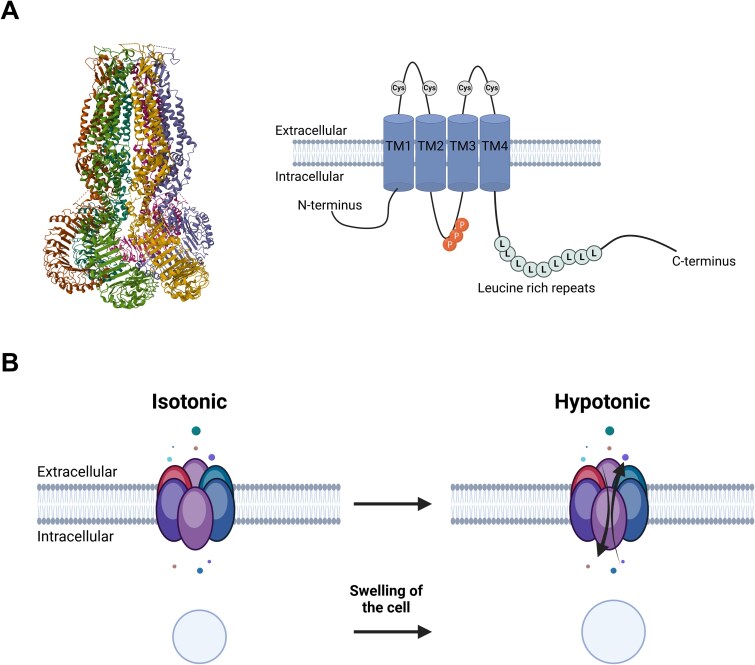
(A) Left: crystal structure of a hexameric LRCC8A channel (PDB: 8DXN); right: schematic drawing of an LRRC8 subunit (created with BioRender); TM, transmembrane segment; L, leucine; Cys, cysteine; *P*, phosphorylation site. (B) Schematic drawing of an LRRC8 subunit und isotonic and hypotonic conditions (modified after,^74^ created with BioRender).

Volume-regulated anion channels are essential for the cellular response to volume changes. These channels are activated in response to hypotonic cell swelling and facilitate the efflux of chloride ions via *I*_Cl,Swell_ and organic osmolytes, thereby restoring cell volume. Volume-regulated anion channels are hexameric channels with LRRC8A (SWELL 1) being the essential subunit^70^ (see Figure 3). They may form homomeric (ie, solely consisting of LRRC8A subunits) and, more commonly, heteromeric channels. In total, there are 5 LRRC8 subunits (A-E) encoded by the *LRRC8* gene, all of which have 4 transmembrane domains and a characteristic C-terminal end with 17 leucine rich repeats. Volume-regulated anion channels are ubiquitously expressed and play pivotal roles in various physiological processes, including cell proliferation, apoptosis, and signal transduction. Zhang and colleagues recently demonstrated expression of LRRC8A in human bone.^71^

While data on VRACs in osteoblasts and osteoclasts are sparse, osteoblasts undergo significant volume changes during mechanical adaptation, suggesting VRAC involvement.^72^ Moreover, in a mouse model of VRAC inhibition, skeletal muscle myoblast differentiation was significantly inhibited by plasma membrane hyperpolarization during early differentiation stages,^73^ suggesting potential VRAC involvement in other myeloid cell lines, including osteoblasts and osteoclasts.

Macrophages utilize cell volume regulation as a mechanism to promote and amplify inflammatory responses. In these cells, VRACs activate the cGAS-cGAMP-STING pathway, thereby inducing type I interferon responses,^75^ which have recently been implicated in age-related bone loss.^76^

LRRC8A, the obligatory VRAC subunit, also appears to be critical for canonical NLRP3 inflammasome activation, including in inflammatory joint disease.^77^ Wu et al. demonstrated that BMDMs from Lrrc8a KO mice exhibit markedly reduced NLRP3 activation—independent of the priming process—accompanied by decreased production of IL-1β and TNF-α, 2 cytokines with well-established effects on bone remodeling.^78^ While potassium efflux is a known prerequisite for NLRP3 activation and can be triggered by potassium-free medium,^79^ BMDMs lacking LRRC8A display unaltered potassium efflux under such conditions, yet still fail to activate the NLRP3 inflammasome. This finding suggests that VRAC functions downstream of potassium efflux in the NLRP3 signaling cascade.^80^

Proinflammatory cytokines, such as TNF-α and IL-1β, can also activate VRAC currents under isovolumetric conditions via cGAS-dependent mechanisms and distinct LRRC8 subunit combinations, notably LRRC8A and LRRC8E.^81^ In vascular smooth muscle cells, TNF-α-induced VRAC activation has been linked to NADPH oxidase 1 (NOX1)-mediated reactive oxygen species generation, providing a mechanistic rationale for the anti-inflammatory effects observed upon VRAC inhibition.^82^

Volume-regulated anion channels are promising but understudied candidates in bone biology. Their established roles in immune activation and mechanical adaptation support their potential relevance in osteoporosis, warranting more extensive targeted bone-specific research.

## Therapeutic potential

Mechanosensitive ion channels may represent a novel therapeutic axis for treatment of osteoporosis by targeting both bone-forming and immune-regulatory pathways. They act as essential mechanosensors and influence the activity of osteoblasts, osteoclasts, and macrophages. Mechanotransduction is a fundamental aspect of bone remodeling, and pharmacological modulation, as well as lifestyle changes, such as increased physical activity, are an integral component of both primary and secondary osteoporosis prevention.

Additionally, mechanosensitive ion channels are involved in regulating several inflammatory pathways. For example, they play a significant role in shaping macrophage phenotype. Depending on their polarization (ie, the pro-inflammatory M1 type vs the anti-inflammatory M2 type), macrophages perform a multitude of functions in bone metabolism and have a substantial impact on bone remodeling. The NLRP3 inflammasome in macrophages is a key component of the inflammatory response. Studies have highlighted its crucial role in bone metabolism. In ovariectomized mice, NLRP3 depletion prevents bone loss.^83^ Consistent with this, NLRP3 activation has been linked to abnormal skeletal development and osteoporosis in mice.^84^ In postmenopausal women, IL-1β is a major factor in bone loss associated with estrogen deficiency.^85^ NLRP3-induced IL-1β production appears to be essential in postmenopausal bone loss. Furthermore, the NLRP3 inflammasome has been implicated in age-related bone loss, as NLRP3-deficient mice exhibit increased bone volume with age compared to wild-type littermates.^86^

Clinically, several IL-1 pathway inhibitors, such as Anakinra (an IL-1 receptor antagonist), have already been approved for treating inflammatory diseases like rheumatoid arthritis. More recently, inflammasome inhibitors, such as VX-765 and MCC950, have entered phase II clinical trials for treatment of epilepsy and rheumatoid arthritis, respectively. However, the results so far have been mixed, showing either limited efficacy or undesirable side effects.^87^ Targeting ion channels to modulate NLRP3 activity presents a novel therapeutic opportunity to limit the chronic inflammatory component of osteoporosis that has yet to be explored. TREK-1, VRAC, and Piezo channels, all of which are sensitive to mechanical stress, represent intriguing targets in this context.

So far, there have been no studies on the significance of ion channels in Osteomacs. Based on the results of the studies on BMDMs presented here and the now well-known central role of Osteomacs in bone metabolism—particularly their ability to influence the inflammatory microenvironment in bone independent of circulating cells—mechanosensitive ion channels may constitute an innovative approach for the development of new therapeutic options specifically targeting Osteomacs.

The K_2P_ channel TREK-1 is expressed in human bone and is essential for inflammasome activation. Furthermore, inhibition of TREK-1 in osteoblasts may decrease the activity of the NLRP3 inflammasome, as well as the production and release of IL-1 β and cleaved Caspase-1.^28^

Volume-regulated anion channels have gained increased recognition for their role in adaptive and innate immune responses, after they were first identified in a patient with defective B-cell development.^88^ In macrophages, VRACs activate the cGAS-cGAMP-STING axis and play an important role in NLRP3 inflammasome activation, both of which are significant mechanisms in bone remodeling. Additionally, osteoblasts undergo substantial volume changes during adaptation to mechanical stress, highlighting VRAC’s potential contribution to osteoblast function and bone homeostasis. However, VRAC’s precise role in osteoporosis pathophysiology remains unexplored. Nonetheless, VRAC inhibition holds promise as a therapeutic target for inflammatory bone loss associated with aging, postmenopausal osteoporosis, and rheumatic diseases.

Nevertheless, it should be noted that functional data for VRAC and K_2P_ channels in the context of bone biology are still limited. Most available studies rely on data from immortalized cell lines such as MG-63 osteoblast-like cells, which may not fully capture the behavior of primary, disease-specific human bone cells. While mechanistic plausibility and findings from related cell types support a potential role for these channels in bone metabolism, these observations require validation in primary human cells, disease-relevant models, and in vivo studies.

Piezo channels are emerging as a promising therapeutic target in osteoporosis treatment, and, at present, there is greater experimental evidence available in this context than for the other ion channels discussed here. Activation of Piezo1 by Yoda1 has been shown to increase bone mass in mouse models.^56^ Piezo1 KO in skeletal progenitor cells or osteoblasts led to severe osteoporosis in mice.^62^ In macrophages, Piezo1 activation induces a pro-inflammatory polarization and activates NLRP3, which likely contributes to bone resorption, although this has not been specifically tested in osteoporosis. Piezo1, therefore, represents a compelling therapeutic target for treating and potentially preventing osteoporosis. Further studies are needed to determine the most effective methods for targeting Piezo channels in osteoporotic bone. Disease-specific cell culture and in vivo experiments, as well as cell type-specific inhibition or activation, should be considered. Myeloid-specific ablation of Piezo1/2 in mice has demonstrated protective effects against gouty arthritis,^65^ but its effect on osteoporosis remains unexamined.

Of note, beyond their roles in adult bone homeostasis, mechanosensitive ion channels may also have important developmental functions. Piezo1, for example, is required for skeletal development in mice, with deletion leading to impaired bone formation during growth phases.

Moreover, the ion channels discussed in this article also appear to play important roles in chondrocytes. Recent studies have shown that Piezo channels are functionally relevant in chondrocytes, as chondrocyte-specific inactivation of Piezo1 led to impaired endochondral ossification and a milder course of osteoarthritis.^89^ In addition, cell volume and hydration of chondrocytes are crucial for both cartilage development and the pathogenesis of osteoarthritis. In this context, VRAC channels were recently shown to play an important role in shaping cartilage morphology in a mouse model.^90^ Functional expression of TREK-1 has also been detected in chondrocytes, and a potential role for this channel in the adaptation of cartilage to mechanical loading has been proposed.^91^ Although the role of these ion channels in chondrocytes is not the primary focus of this perspective, we consider it important to briefly mention these findings, as they underscore the broader relevance of these channels in musculoskeletal diseases beyond osteoporosis.

Most of the data available so far pertains to the relevance of ion channels in osteoblasts, osteocytes and macrophages, while information on the significance of ion channels in osteoclasts is more limited. However, osteoclasts, in particular, rely on an acidic environment for their resorptive activities and must withstand significant osmotic pressures, highlighting the critical importance of ion channels for osteoclast homeostasis and functionality in bone remodeling. Beyond various ATPases, chloride channels, and chloride-potassium cotransporters, little is known about the role of the aforementioned ion channel classes in osteoclasts. The K_2P_ channel TWIK-1 is one notable exception. For this channel, an inhibitory effect on osteoclastogenesis via modulation of the JNK-NFATc1 signaling cascade has been described.^27^ Therefore, we believe that the ion channel families discussed here should be investigated more extensively in osteoclasts in future studies to better understand their role in altered bone homeostasis in osteoporosis.

While the majority of mechanistic insights into K_2P_, Piezo, and VRAC channels in bone biology stem from in vitro experiments, these findings should be interpreted with caution. Cell lines and isolated primary cultures provide valuable mechanistic information but may not fully replicate the complex microenvironment of bone tissue, which includes dynamic mechanical loading, three-dimensional matrix interactions, and systemic hormonal regulation. Further validation in in vivo models, ideally combined with human patient-derived data, will be essential to confirm translational relevance.

Furthermore, given the wide distribution and diverse functions of these channels, achieving a bone-specific influence remains challenging. Cell type-specific genetic models offer an effective way to define their roles, particularly for VRAC and K_2P_ channels, where functional data, especially in osteoclasts and Osteomacs, are still lacking.

## Data Availability

No new data were generated or analyzed in support of this research.
